# Truncated Electrochemical Aptasensor with Enhanced Antifouling Capability for Highly Sensitive Serotonin Detection

**DOI:** 10.3390/bios13090881

**Published:** 2023-09-11

**Authors:** Ziheng Hu, Ruifeng Zhu, Gabriela Figueroa-Miranda, Lei Zhou, Lingyan Feng, Andreas Offenhäusser, Dirk Mayer

**Affiliations:** 1 Institute of Biological Information Processing, Bioelectronics (IBI-3), Forschungszentrum Jülich GmbH, 52428 Jülich, Germany; z.hu@fz-juelich.de (Z.H.); r.zhu@fz-juelich.de (R.Z.); g.figueroa.miranda@fz-juelich.de (G.F.-M.); leizhou1@outlook.com (L.Z.); a.offenhaeusser@fz-juelich.de (A.O.); 2 Faculty I, RWTH Aachen University, 52062 Aachen, Germany; 3 Department of Materials Genome Institute, and Department of Chemistry, College of Science, Shanghai University, Shanghai 200444, China; lingyanfeng@t.shu.edu.cn

**Keywords:** aptamer, neurotransmitter, polyethylene glycol, antifouling, electrochemistry

## Abstract

Accurate determination of serotonin (ST) provides insight into neurological processes and enables applications in clinical diagnostics of brain diseases. Herein, we present an electrochemical aptasensor based on truncated DNA aptamers and a polyethylene glycol (PEG) molecule-functionalized sensing interface for highly sensitive and selective ST detection. The truncated aptamers have a small size and adopt a stable stem-loop configuration, which improves the accessibility of the aptamer for the analyte and enhances the sensitivity of the aptasensor. Upon target binding, these aptamers perform a conformational change, leading to a variation in the Faraday current of the redox tag, which was recorded by square wave voltammetry (SWV). Using PEG as blocking molecules minimizes nonspecific adsorption of other interfering molecules and thus endows an enhanced antifouling ability. The proposed electrochemical aptamer sensor showed a wide range of detection lasting from 0.1 nM to 1000 nM with a low limit of detection of 0.14 nM. Owing to the unique properties of aptamer receptors, the aptasensor also exhibits high selectivity and stability. Furthermore, with the reduced unspecific adsorption, assaying of ST in human serum and artificial cerebrospinal fluid (aCSF) showed excellent performance. The reported strategy of utilizing antifouling PEG describes a novel approach to building antifouling aptasensors and holds great potential for neurochemical investigations and clinical diagnosis.

## 1. Introduction

Serotonin (ST), as a catecholamine neurotransmitter, plays a critical role in mediating various biological and physiological functions in different parts of the human body [1,2]. Normally, ST appears in blood at a concentration of around 0.6–2 µM, 0.03–0.13 µM in urine, and approximately 10 nM in cerebrospinal fluid (CSF) [3,4]. In the central nervous system (CNS), ST is engaged in different regulatory processes including mood, sleep, emesis, sexuality, and appetite [5,6]. Abnormal expression of ST has been linked to several neurological disorders, such as depression, Alzheimer’s, and Parkinson’s disease [7,8]. However, only 5% of the overall ST content in the body is found within the CNS. The majority of ST is found peripherally, in blood and the gut. Raised ST levels in the plasma are associated with several gastrointestinal illnesses such as serotoninergic malfunctions [9,10]. Recently, the interdependence of gastrointestinal and brain health has attracted considerable scientific interest [11]. Accordingly, ST is considered a potential biomarker in medical diagnosis for a variety of illnesses and thus an attractive target for the development of biosensors.

To date, numerous methods have been established for the analysis of ST, including high-pressure liquid chromatography (HPLC), fluorescence, and mass spectroscopy [12,13,14,15]. Although these approaches exhibit high selectivity and precision in ST detection, they nevertheless require the use of expensive instruments and complex sample preparation procedures, making them unsuitable for routine tests and point-of-care examinations. By utilizing the electroactive properties of ST, electrochemical methods are commonly employed due to their simplicity, fast response, high sensitivity, and repeatability [16,17,18,19]. In the past few years, several electrochemical sensors with nanomaterial-modified surfaces, which enhanced their electrocatalytic activity, have been developed and demonstrated an improved performance for the ST detection [20,21,22,23]. However, the coexistence of interfering molecules with similar oxidation potential to ST such as dopamine (DA) and ascorbic acid (AA) cause selectivity issues due to overlapping redox responses [24,25]. Furthermore, despite recent progress in surface modification methods, the strong electrode fouling due to ST oxidation products leads to vulnerable fouling issues and affects the sensing performance [26]. Thus, further advancements are required to develop simple and reliable analytical methods for ST determination.

Recently, aptamers for ST detection have been introduced as receptor molecules in the electrochemical sensors [27,28]. These receptors were selected from libraries of single-stranded nucleic acid oligomers via a process known as systematic evolution of ligands by exponential enrichment (SELEX). With the properties of high binding affinity, selectivity, and stability aptamers have the potential to revolve neurotransmitter analysis [29,30]. Recently, Nakatsuka et al. reported the first aptamer against ST with high affinity (K_d_ = 30 nM) and used it for ST analysis by field-effect transistors and nanopipette devices [31,32,33]. In our previous work, we applied this aptamer for the development of a first-generation electrochemical aptasensor and achieved fM-level detection limits for ST [34]. However, the original aptamer strand possesses a long chain length with a large stem-loop structure, which impairs its sensitivity during electrochemical detection. In particular, the interaction between neighboring strands can affect the sensor sensitivity during the binding of the small molecule target [35,36]. Therefore, an elaborated nano-fabrication transducer or specific redox molecule labeling had to be used to facilitate sensitive ST monitoring with this aptamer [37,38]. Therefore, there is a remaining need for the optimization of the ST aptamer sequence to enable simple sensor fabrication and versatile deployment.

Besides the receptor molecules, the biofouling effects also lead to decreased sensing performances of electrochemical aptamer-based (E-AB) sensors when detecting targets in real complex samples (e.g., human plasma, blood serum, and cell pulverization) [39]. The undesired nonspecific adsorption, especially the adsorption of matrix proteins on the electrode interfaces, can impair the selectivity and sensitivity of the device, thereby decreasing the accuracy and reproducibility of the detected signals [40]. To overcome this issue, aptamer molecules are immobilized together with blocking molecules to build a receptor layer with antifouling properties. At present, various antifouling materials, including zwitterionic molecules, peptides, and alkanethiols, have been proposed and widely utilized for the fabrication of aptasensors [41,42,43]. Although in general these materials show biofouling resistance and enhance selectivity in complex environments, their synthesis and immobilization processes are partially tedious and time consuming. Polyethylene glycol (PEG), a biocompatible and very hydrophilic coating molecule, is commonly regarded as the “gold standard” of anti-biofouling polymers to prevent nonspecific protein adsorption [44,45,46]. The entropic effect of steric repulsion, which is connected with the unfavorable variation of free energy linked to confinement and dehydration of the polymer chains, is considered a reason for the prevention of protein adsorption on PEG-modified surfaces [47,48]. Recently, our group utilized a PEG-mediated coating interface for an electrochemical impedance aptasensor and obtained promising results for Malaria biomarker detection in human serum [49].

**Scheme 1:**
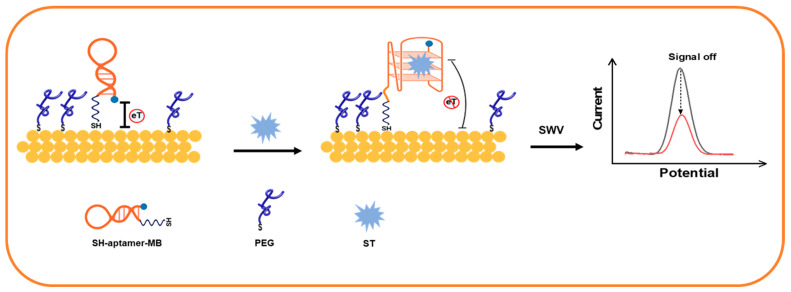
The schematic illustration of the working principle of the electrochemical aptasensor for serotonin detection with PEG backfill.

In this work, we propose an electrochemical sensor utilizing a truncated ST aptamer and PEG as blocking molecules. As depicted in Scheme 1, the thiol-tagged aptamer strand was tethered to the gold electrode (AuE) through typical Au–S bonding forming a mixed self-assembled monolayer together with the PEG blocking agent. To facilitate charge-transfer-based detection, methylene blue (MB) was attached as a redox tag at the distal end of the aptamer for signal reporting. The surface-associated binding processes were monitored by SWV, which facilitated signal recording with a low noise level. In the absence of analyte ST, the probe adopts a stem-loop configuration that clamps the attached redox tag in proximity to the electrode, which causes a high charge transfer signal. During target binding, the aptamer performs a conformational rearrangement resulting in a G-quadruplex complex that positions the MB tag away from the electrode, impending the electron transfer and leading to a distinct decrease in the redox current. By monitoring the variation of the Faraday current, a quantitative analysis of ST was achieved.

## 2. Materials and Methods

### 2.1. Regents

The used HPLC-purified DNA aptamers were obtained from FRIZ Biochem (Neuried, Germany). The sequences were as follows:

S1: 5′-OH-(CH_2_)_6_-S-S-(CH_2_)_6_- CGA CTG GTA GGC AGA TAG GGG AAG CTG ATT CGA TGC GTG GGT CG -MB-3′

S2: 5′-OH-(CH_2_)_6_-S-S-(CH_2_)_6_- TAG GCA GAT AGG GGA AGC TGA TTC GAT GCG TG-MB-3′

S3: 5′-OH-(CH_2_)_6_-S-S-(CH_2_)_6_- GCA GAT AGG GGA AGC TGA TTC GAT GC-MB-3′

The aptamer concentration was obtained by recording the absorbance at a 260 nm wavelength with UV/vis spectroscopy (DS-11 Series Spectrophotometer/Fluorometer, DeNovix Inc., Wilmington, DE, USA). All tested solutions were prepared with Milli-Q water produced by a Milli-Q ultrapure water system (18.25 MΩ cm, Gradient A10, Merck Millipore, Burlington, MA, USA). Monofunctional methoxy-polyethylene glycol thiol (PEG, 2 kDa), tris-(2-carboxyethyl) phosphine hydrochloride (TCEP), 6-mercaptho-1-hexanol (MCH), potassium chloride, magnesium chloride, potassium ferrocyanide (K_4_[Fe(CN)_6_]), sodium chloride, potassium ferricyanide (K_3_[Fe(CN)_6_]), and human serum from human male AB plasma were obtained from Sigma-Aldrich Chemie GmbH (Darmstadt, Germany). Ethanol and isopropanol were ordered from Merck (Darmstadt, Germany).

### 2.2. Electrode Cleaning, Aptasensor Preparation, and Target Detection

Before the aptamer modification, the blank AuE (ø 2 mm, Shanghai Chenhua CHI) was polished with alumina powder (0.3 and 0.05 μm) and then sonicated in MilliQ water to remove polishing agent residuals. Afterwards, an electrochemical cleaning procedure was applied following the protocol described previously [50]. The active electrode area was obtained from cyclic voltammetry (CV) scans recorded in 0.05 M H_2_SO_4_ [51].

Before any modification, the thiolated aptamer was mixed with 10 mM TCEP at room temperature for 60 min to split the disulfide bonds. Then, the cleaned AuE was immersed in high salt Tris-HCl buffer (10 mM Tris, 1.5 M NaCl, 1 mM MgCl_2_, pH 7.4) containing 0.5 μM aptamer overnight. A self-assembled monolayer formed during this time via thiol–gold binding between the thiolated molecules and the Au surface. Subsequently, the aptamer-modified electrode was incubated in an aqueous solution with 1 mg/mL PEG for various indicated times. Lastly, the modified AuE was rinsed with the low salt PBS buffer to remove non-covalently bound molecules and stored in the same buffer before the measurements. Artificial cerebrospinal fluid (aCSF) was made by mixing 150 mM NaCl, 1.4 mM CaCl_2_·2H_2_O, 3.0 mM KCl, 1 mM NaH_2_PO_4_, and 0.8 mM MgCl_2_·6H_2_O in Milli-Q water. To demonstrate the versatility of our aptasensor, the aCSF was spiked with 45 mg dL^−1^ human serum albumin to imitate its native analogous liquor. The diluted human serum solution was prepared by mixing the purchased human serum with the low salt phosphate-buffered saline (PBS, 137 mM NaCl, 2.7 mM KCl, 10 mM Na_2_HPO_4_, 1.8 mM NaH_2_PO_4_, 2 mM MgCl_2_, pH 7.4) based on the volumetric ratio. All experiments were carried out in triplicate. The statistical standard deviation (*SD*) was calculated as:

$$SD=\sqrt{\frac{\sum {\left(X-M\right)}^{2}}{n-1}}$$

where *X* refers to the individual data points, *M* is the mean, and *n* the number of data points.

### 2.3. Electrochemical Measurements

All electrochemical data were obtained from an Autolab potentiostat/galvanostat PGSTAT302 (Eco Chemie, Utrecht, The Netherlands) with a three-electrode system including a AuE working electrode, saturated Ag/AgCl reference electrode, and a platinum wire counter electrode. SWV measurements were performed in low salt PBS buffer at room temperature (25 °C) via potential scans between −0.5 and 0 V and a pulse frequency of 10 Hz. This technique was utilized since it resulted in the largest signals for ST detection. Electrochemical impedance spectroscopy (EIS) and CV measurements were performed to characterize the electrode modification with a 5 mM solution of 1:1 ferro/ferricyanide dissolved in PBS. CV measurements were recorded in a potential window lasting from −0.2 V to 0.6 V with a scan rate of 100 mV/s. Electrochemical impedance spectroscopy measurements were conducted at a potential bias of 0.2 V within a frequency range from 1 Hz to 10 kHz with an amplitude of 10 mV.

Additionally, the surface aptamer density was determined on an electrochemical workstation CHI1030B (Austin, USA) by chronocoulometric measurements of potential steps between 0.2 and −0.5 V in 10 mM hexaammineruthenium (III) chloride (RuHex) containing 10 mM Tris-HCl buffer (pH 7.4).

### 2.4. QCM-D Measurements

The quartz crystal microbalance measurements with dissipation monitoring (QCM-D, Västra Frölunda, Schweden) were used to monitor the mass change during each immobilization step. Before the measurements, all QCM-D components, including the microfluidic flow cell and gold sensors were cleaned as suggested by the supplier. Therefore, the QCM gold samples were treated in an oxygen plasma at an O_2_ pressure of 0.5 mbar for 3 min with a power of 50%. Subsequently, the gold oxide that was formed during the plasma treatment was removed by incubating the sample in ethanol for 30 min. After the cleaning, the QCM-D cell was mounted and the sensor was placed onto the QCM-D module. Afterwards, the buffer solution was rinsed through the cell driven by a pump until the signal stabilized with a frequency shift below 0.2 Hz over 10 min. For the actual experiment, the different molecule solutions were consecutively flushed over the sensor followed by a rinsing with PBS buffer.

### 2.5. Measurements by Atomic Force Microscopy

Atomic force microscopy (AFM) images were recorded using a Nanoscope Multimode 8 microscope (Bruker). The microscope was equipped with a piezoelectric E-scanner and aluminum back-coated Si cantilevers from Bruker (OTESPA-R3) were utilized for imaging with a resonant frequency range of 115–300 kHz. The 1 × 1 μm images were recorded with 512 × 512 pixels at a scan rate of 1 Hz. An ultrasmooth polished gold (111) single-crystal disk was employed as a model electrode surface to be able to identify the step-wise immobilization of aptamer and blocking molecules. At first, a coarse cleaning of the single crystal was performed by rinsing it in ethanol, isopropanol, and Milli-Q water. Subsequently, the crystal was flame-treated in a hydrogen flame for 10 min. After cooling to room temperature under an argon atmosphere, aptamer immobilization and analyte detection experiments were performed as described in Section 2.2.

## 3. Results and Discussion

### 3.1. Optimization of the Biosensor Fabrication

**Figure 1:**
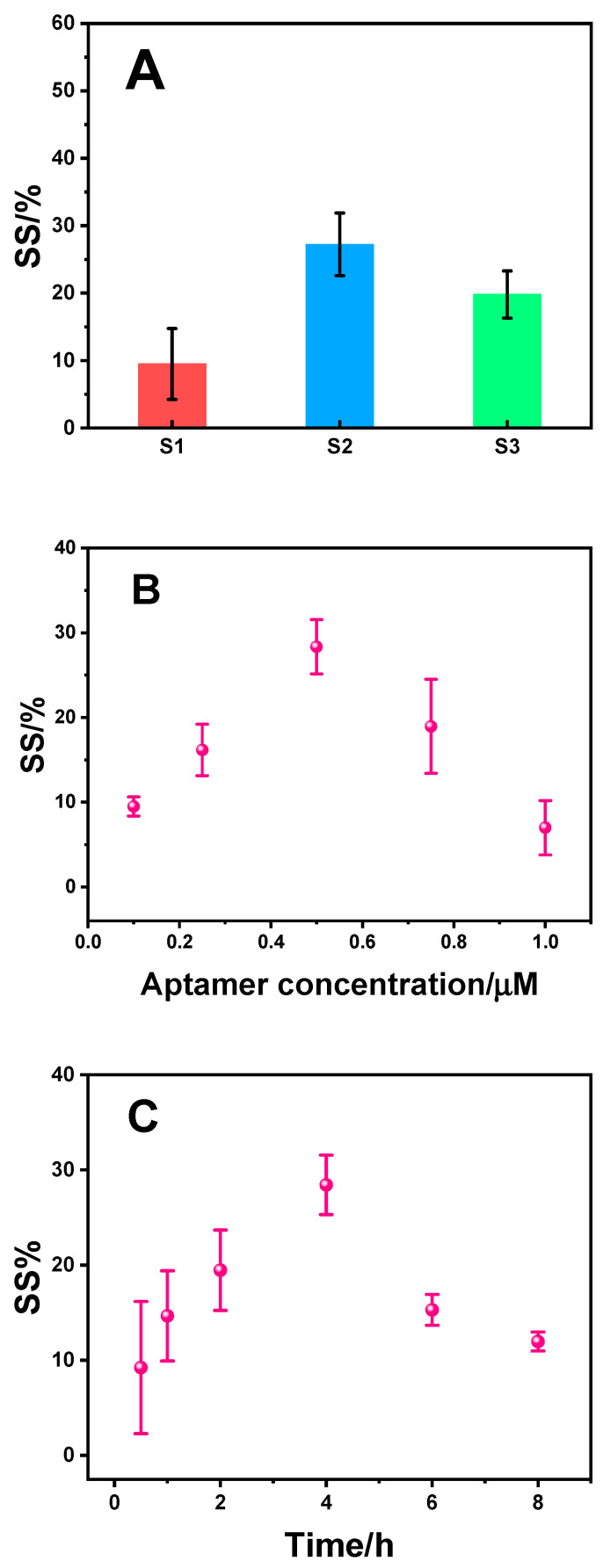
(**A**) Comparison of three different aptamer lengths with respect to the signal change (SS%) induced by adding 10 nM ST. (**B**) Optimization of S2 aptamer concentration and (**C**) optimization of blocking PEG incubation time (error bar means data standard deviation (SD), *n* = 3).

The aptamer receptor used in this work was selected by Nakatsuka et al. and contains a stem-loop structure, which results in signal-off characteristics for E-AB sensors. The greatest advantage of this stem-loop-containing aptamer is the strong conformational change that is induced during the binding of the analyte. Furthermore, stem-loop structures effectively reduce unspecific charge transfer because of the reduced degree of freedom of the aptamer immobilized on the electrode surface. To obtain a high signal response, the stem-loop configuration of the aptamer was optimized by sequence truncation. Therefore, the relative signal suppression (SS) of the MB Faraday current was recorded as the sensor signal according to (*I − I*_0_)/*I*_0_ (%), where *I* and *I*_0_ are peak faradic currents from the terminal redox molecule of the aptamer with and without target binding, respectively. Here, the SS of the originally selected oligonucleotide sequence (S1) and two aptamers with different lengths (S2 and S3) truncated from the original aptamer were tested after target binding. Their responses to the same concentration of ST (10 nM) are shown in Figure 1A. The AuE modified with the S2 strand (32 bp) exhibited the biggest signal decrease of about 27.2 ± 4.6%, whereas the SS% of S1 (44 bp) and S3 (26 bp) were 19.8 ± 3.5% and 9.5 ± 5.2%, respectively. The signal difference can be attributed to differences in the stem length of the aptamer stem-loop structure, which is a key parameter for the charge transfer response from redox probes located at the terminal end of the aptamer. It has been reported that the hairpin-like conformation will not open or open too easily if the stem is too long or short, respectively, thus affecting the signal response upon target binding [36,52]. Compared to the S2 strand, the S1 strand owns a longer stem length which means strong competition between stem formation and target binding. Consequently, the stem structure is preserved, even in the presence of the analyte and the distance between the electrode surface and the redox probe does not significantly change. For strand S3, some binding regions necessary for the interaction have been ousted in the truncation process, and a sturdy stem-loop structure could not form because of the short stem length after truncation. The majority of the aptamer molecules just undergo a small conformational change after target binding [53]. In contrast, the S2 strand adopts a stable and proper stem-loop configuration, which is crucial for the binding capability of the aptamers. Moreover, the smaller size of the S2 strand not only lowers the synthesis cost but also improves the accessibility of the target to the aptamer, which further enhances the sensor sensitivity [54]. Therefore, we chose the S2 strand with optimal stem length and large signal change for the subsequent experiments.

To ensure optimal sensor performance, the S2 aptamer density on the sensor surface was tuned by optimizing the aptamer concentration during its binding to the electrode, Figure 1B. The data show that the SS% first increased with the increasing aptamer concentration, followed by a strong decrease. The maximum SS% was obtained at 0.5 μM aptamer and its surface coverage was calculated to 1.05 × 10^12^ molecules/cm^2^ by chronocoulometric measurements, Figure S1. The reason for the low signal for the low aptamer concentration is the small surface density and thus the small number of binding events. For high aptamer concentrations, electrostatic repulsion between neighboring negatively charged aptamer molecules can prevent the analyte binding. Furthermore, the dense aptamer packing on the surface does not provide enough space for the aptamer to fold into the 3D conformation required for target binding [55]. Therefore, a 0.5 μM aptamer concentration was used during our sensor fabrication.

In addition, the incubation condition of the blocking molecule can affect the sensor performance. Therefore, the composition of the mixed S2 aptamer-PEG receptor layer was optimized by varying the PEG incubation time, Figure 1C. For short PEG incubation times, the SS% was low due to the low density of the blocking molecules. There, some uncovered electrode area remained after aptamer modification and the aptamer molecules lay down and interacted un-specifically with the electrode surface, which impaired the recognition of the analyte. Furthermore, the mixed monolayer with low PEG density could not effectively prevent antifouling and corresponding protein adsorption [49]. As the incubation time increased, latterly PEG molecules occupied the bare sites of the electrode and cleaved the interactions between lying aptamers and AuE leading to upright standing molecules, making target binding more favorable. Thus, the SS% value rose with increasing PEG incubation times and reached its maximum at 4 h. A long incubation forms a high-density PEG layer, which leads to a complete blocking of the analyte binding and an associated signal drop since the aptamer molecules require a certain area on the transducer surface for its 3D conformational adaptation to its target. Interestingly, the optimal PEG incubation time of 4 h for the serotonin sensor is shorter than the previously reported 7 h found for an aptasensor for malaria biomarker indicating that the PEG incubation time is either dependent on the aptamer or on the analyte size.

Finally, to elucidate the dependence of ST binding to its aptamer on the analyte incubation time, the SS% was recorded for different incubation times from 10 to 50 min, Figure S2. A saturation of the aptasensor signal was achieved after 40 min indicating a steady state between the analyte association and dissociation after this time.

### 3.2. The Characterization of the Biosensor Fabrication Process

**Figure 2:**
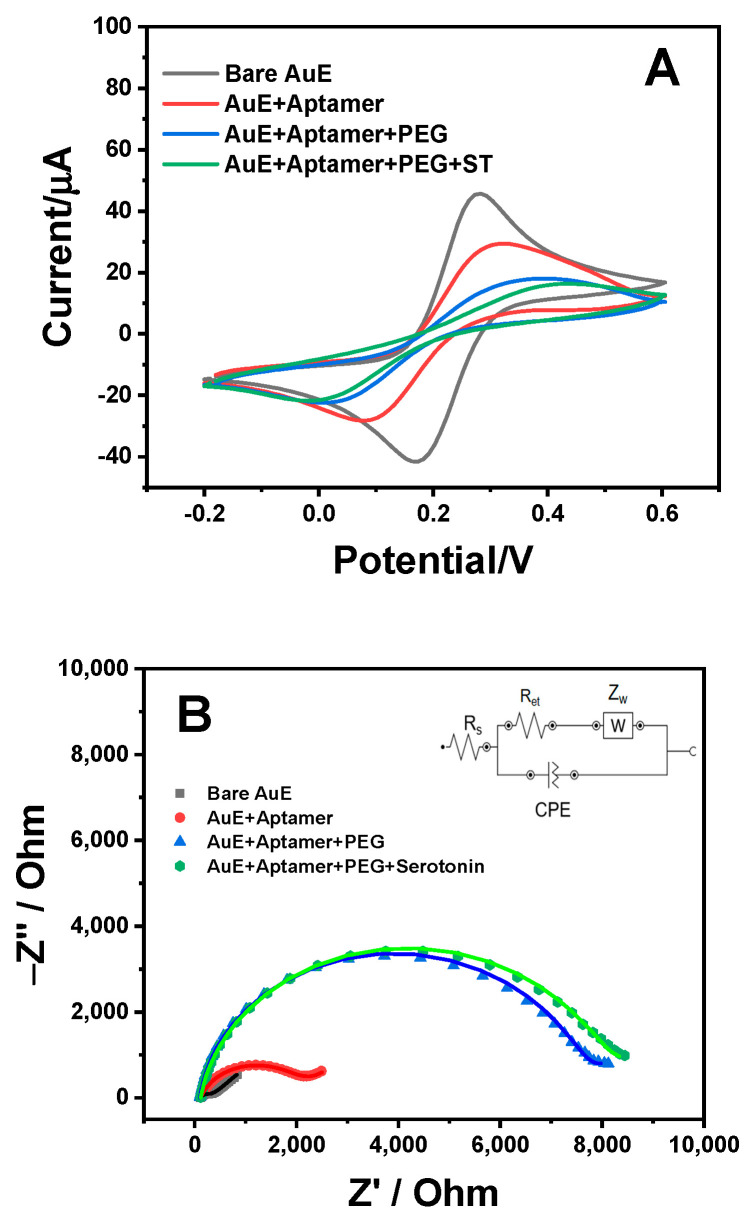
(**A**) CV of stepwise-modified electrode surfaces in PBS buffer with 5 mM [Fe(CN)_6_]^3−/4−^. (**B**) Nyquist plots of the EIS measurements recorded for the same electrodes as in (**A**), where dots represent the experimental data and solid lines represent the fits corresponding to the equivalent circuit shown in the inset.

The AuE electrodes were characterized by CV and EIS after each modification step. The cleaned bare AuE showed a well-defined redox peak for the [Fe(CN)_6_]^3−/4−^ containing the solution, Figure 2A. After the ST aptamer was immobilized, the peak current decreased due to the blocking of the charge transfer by the ssDNA adlayer. The peak current further decreased when PEG was applied on the electrode since those surface sites were then decorated by PEG molecules, which were previously uncovered by aptamers. In the presence of ST, the peak current further decreased due to further hampering the charge transfer between the electroactive [Fe(CN)_6_]^3−/4−^ probe and the electrode surface. Additionally, EIS measurements were employed to evaluate the process of biosensor fabrication, Figure 2B. The diameters of the Nyquist plot semicircles reflect the charge transfer resistance (*R*_et_) values of the electrode after the different preparation steps. As shown in Figure 2B, the *R*_et_ value of bare AuE was as small as 203 Ω, indicating a direct and fast charge transfer process. Upon aptamer assembly on the surface, the *R*_et_ value increased to 2000 Ω, due to the electrostatic repulsion between the redox probes and the negatively charged backbone of the ssDNA molecules. After incubation with 1 mg/mL PEG, an obvious enhancement of the *R*_et_ value to about 7490 Ω was obtained, implying that the direct charge transfer to the modified electrode was well suppressed. Finally, 10 nM target ST was added to the sensor system causing a slight increase in *R*_et_ to 7970 Ω due to the small size of the analyte molecule.

**Figure 3:**
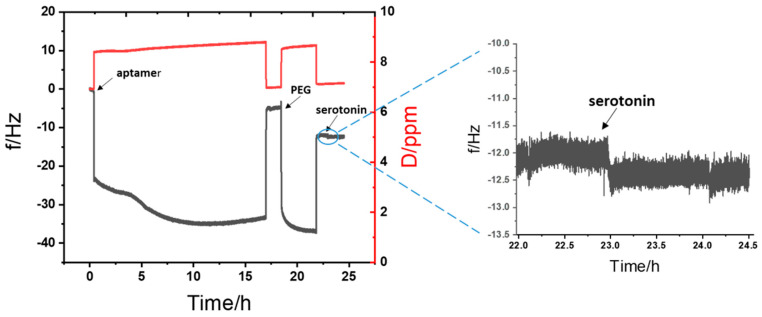
QCM-D data for the immobilization of aptamer and PEG molecules as well as serotonin binding. F stands for frequency and D for dissipation.

Furthermore, the mass change associated with the stepwise immobilization process onto the electrode surface was monitored by using a QCM-D equipped with a fluidic system. This method facilitated the recording of changes in mass load and dissipation during the assembly of a mixed monolayer by the stepwise immobilization of the aptamer receptors and the PEG backfill molecules [56]. According to the Sauerbrey equation [57],

$$\Delta f=\frac{2f_{o}^{2}}{A\sqrt{\rho_{q}\mu_{q}}}\Delta m$$

the frequency change is proportional to the mass increase when the molecules bind to the sensor surface, Figure 3. The purging of 1 µM aptamer solution over the bare Au sensor and subsequent PBS rinsing caused a decrease in the frequency (Δ*f* = 5.05 Hz) indicating the attachment of the aptamer molecules. After flushing the PEG solution (4 h incubation) and rinsing with PBS buffer, a further frequency shift of 7.05 Hz was obtained due to the immobilization of the blocking molecules to the electrode area that was not decorated by aptamer molecules before. Finally, a relatively small frequency shift (Δ*f* = 0.45 Hz) was recorded due to the low molecule weight of ST associated with the binding process of this analyte to aptamer receptors.

**Figure 4:**
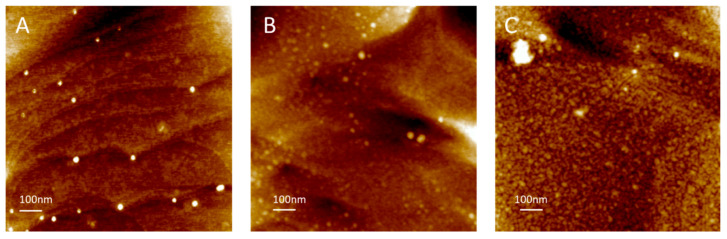
Tapping mode AFM image for a bare gold single crystal (111) surface (**A**), after aptamer immobilization (**B**), and additional PEG binding (**C**). The z scale of (**A**) goes from −1.0 nm to 1.2 nm; the z scale of (**B**) goes from −3.8 to 4.3 nm; the z scale of (**C**) goes from −4.9 to 5.3 nm.

Alterations of the surface morphology corresponding to the aptamer and PEG immobilization were investigated by AFM measurements, Figure 4. A bare gold (111) disk-single crystal with a surface roughness of 0.31 ± 0.01 nm was used as a model surface, which can provide unambiguous imaging of morphological changes during receptor layer formation since it processes atomically flat terraces. After incubation of the aptamer solution, the overall surface roughness rose to 0.96 ± 0.02 nm, indicating that these receptor molecules bound to the surface. However, the AFM image did not show a homogeneous monolayer morphology. It can be assumed that the immobilized aptamer presented an unordered arrangement presumably with molecules partially lying down on the surface and others with upright orientation [49]. The addition of PEG solution can block the remaining free surface area and facilitate the formation of upstanding aptamers surrounded by PEG molecules. Thus, the surface roughness manifested an increment to 1.23 ± 0.01 nm, and the mixed monolayer also indicated common molecular features in AFM images [58,59].

### 3.3. Analytical Performance of the Designed Biosensor

**Figure 5:**
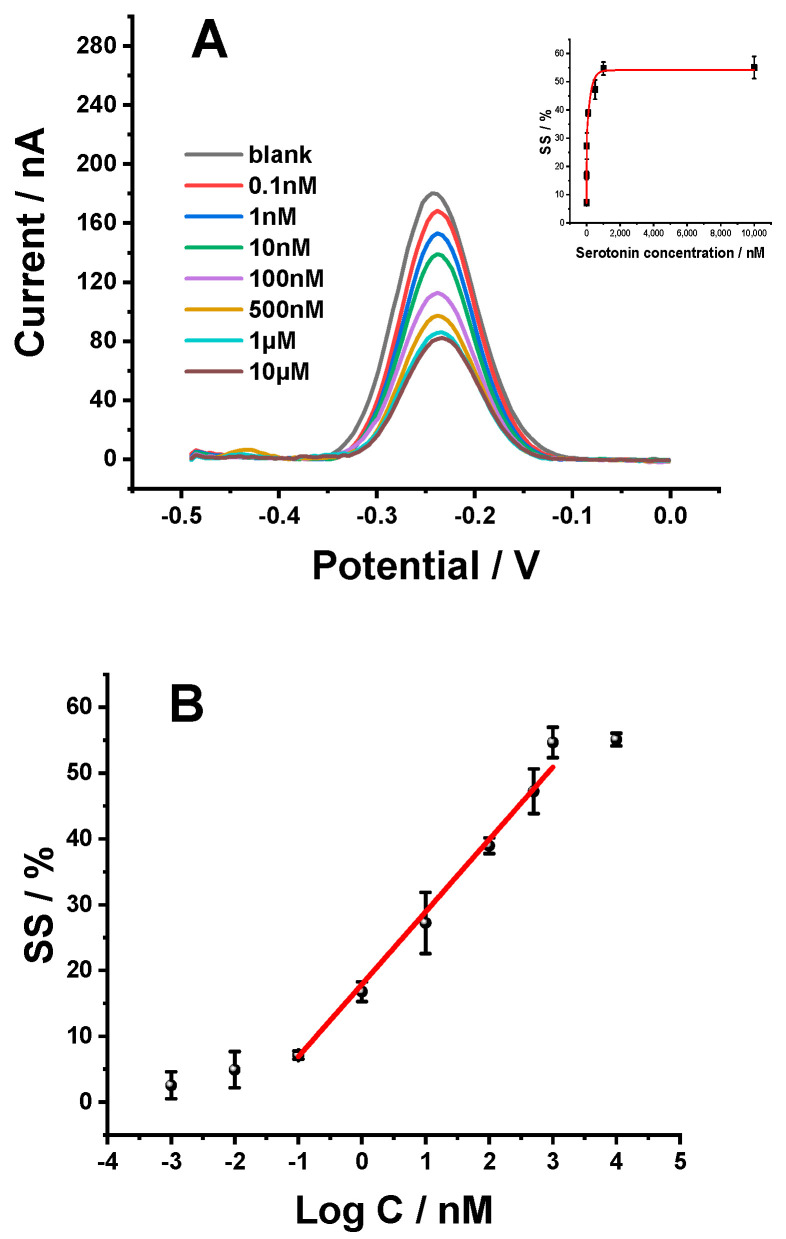
(**A**) SWV curves of the aptasensor with different concentrations of serotonin in PBS buffer (137 mM NaCl, 2.7 mM KCl, 10 mM Na_2_HPO_4_, 1.8 mM NaH_2_PO_4_, 2 mM MgCl_2_, pH 7.4), inset shows the relation of the SS% vs. analyte concentration with a fit by a Langmuir equation. (**B**) Semi-logarithmic presentation of the SS% and the analyte concentration. (Error bar means data standard deviation (SD), *n* = 3).

Under the optimized conditions, the sensitivity and dynamic working range of our aptasensor were investigated by detecting a series of different concentrations of ST in PBS buffer (137 mM NaCl, 2.7 mM KCl, 10 mM Na_2_HPO_4_, 1.8 mM NaH_2_PO_4_, 2 mM MgCl_2_, pH 7.4). As shown in Figure 5A, the SWV peak current decreased with rising target concentrations, due to the binding of ST to the aptamer, which followed a Langmuir–Freundlich equilibrium model (Figure 5A, insert). In addition, the sensor showed a semi-logarithmic concentration-dependence in the range from 0.1 nM to 1 μM and the liner calibration equation was SS (%) = 11.0 log C (nM) + 17.9 with a correlation coefficient of 0.99 (Figure 5B). The limit of detection (LOD) was estimated to be 0.14 nM according to the IUPAC definition: LOD = Mean + 3 SD with Mean as the mean value of the signal gain at the lowest tested concentration from at least three independent experiments and SD as the standard deviation value of the signal gain [60]. The proposed aptasensor exhibited high sensitivity and was able to cover the physiological range of ST. Compared to other existing ST assays (Table S1), our sensor with truncated aptamer showed a competitive analytical performance compared with most previously reported strategies [61,62,63,64,65,66]. More importantly, this method facilitates a simple and universal operation without additional complex modifications.

### 3.4. Selectivity, Regeneration, and Stability

**Figure 6:**
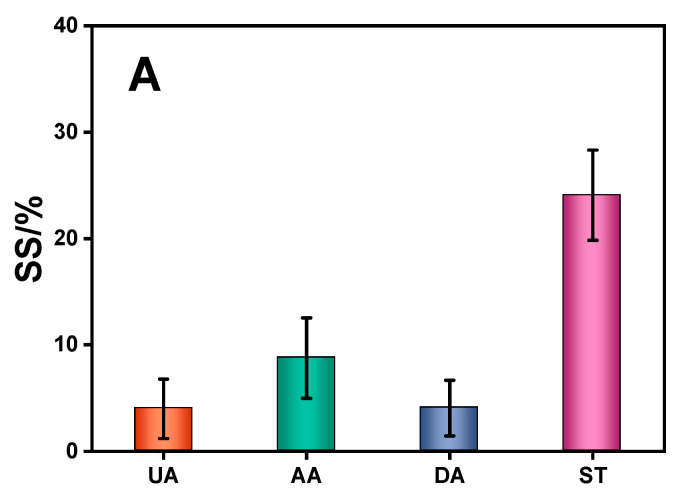

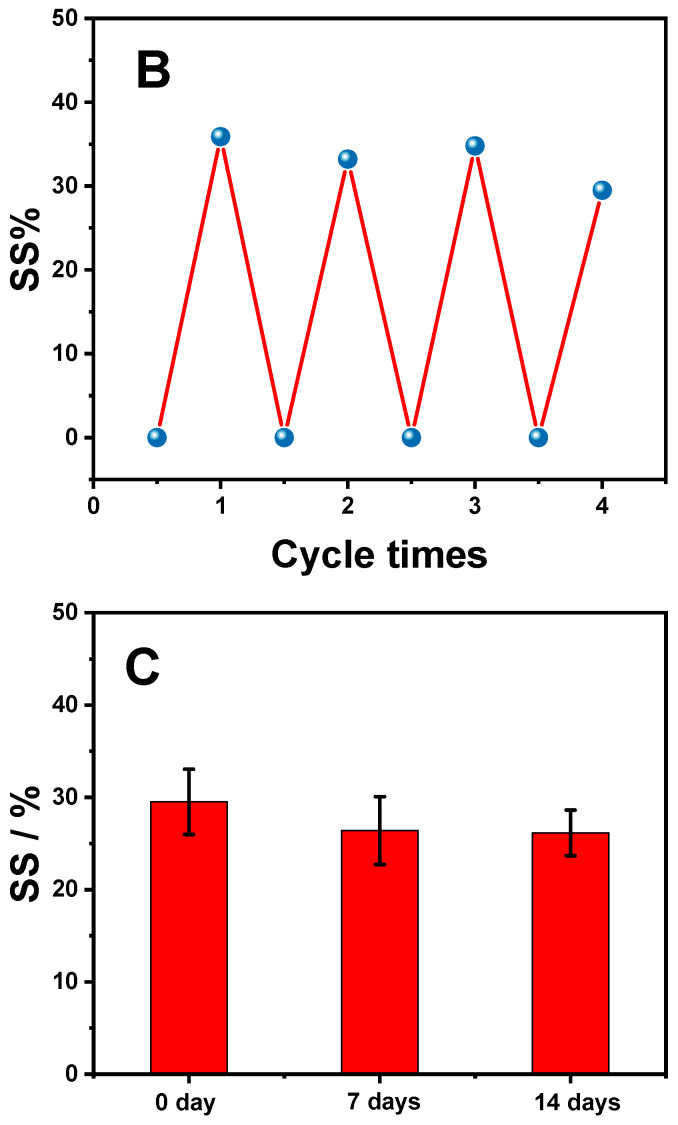
(**A**) Selectivity of the ST aptasensor, where either 1 µM of uric acid (UA), dopamine (DA), ascorbic acid (AA), or 10 nM of ST were added. (**B**) Regeneration of the aptasensor in 10 nM serotonin after a 30 s rinse in 6 M urea over four uses. (**C**) Stability of the aptasensor after 0, 7, and 14 days storage at 4 °C. (Error bar means data standard deviation (SD), *n* = 3).

Apart from the sensitivity, the selectivity of the proposed aptasensor was evaluated towards neurochemicals with similar structured and/or interfering electrochemical activity. The sensor response to 10 nM ST was evaluated in comparison to high concentrations (1 μM) of uric acid (UA), dopamine (DA), and ascorbic acid (AA), Figure 6A. Although, all these molecules possess a similar structure, the sensor with the truncated aptamer S2 displayed comparably low signal variations for interfering molecules, while a distinct SS% increase was observed for ST although its concentration was two orders of magnitude lower concerning the analog molecules. Consequently, the target ST can be easily distinguished from other neurochemicals by this aptasensor due to the high selectivity of the aptamer receptor, which was not impaired by the sequence truncation.

To characterize the reusability and thermo-stability, the regeneration of the aptasensor was examined by consecutive detection to 10 nM ST. After each analyte detection step, the sensor electrode was rinsed with 6 M urea solution for 30 s to dissociate the linkage between aptamer and target and to reactivate the sensor. Owing to the high chemical stability of our sensor system, both thiol-based aptamer and the surface-bound PEG can resist the harsh reactivation treatment and conserve the affinity of the aptamer receptor for its analyte. The analyte binding procedure was repeated several times and the signal change was kept on average over 90% even after four times regeneration, Figure 6B.

Moreover, to further characterize the stability of the detection system, the developed aptasensor was employed to detect ST after 7 and 14 days of storage in a buffer solution at 4 °C, Figure 6C. Compared to a freshly prepared aptasensor, the stored devices maintained around 85% of the original signal, suggesting a considerable shelf life, although the sensor was not yet optimized for long-term storage.

### 3.5. Real Sample Analysis

**Figure 7:**
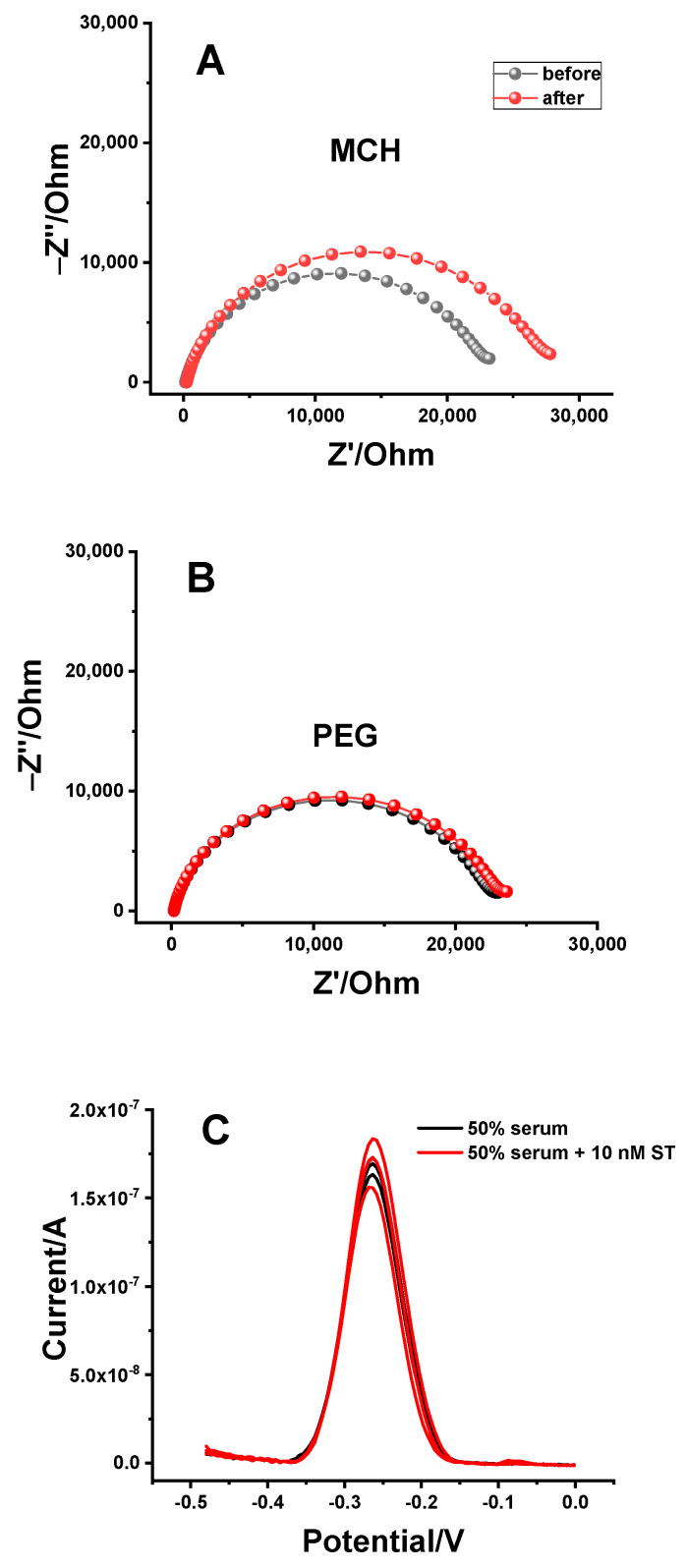
EIS Nyquist plots of Au electrodes demonstrate the non-specific adsorption with (**A**) MCH and (**B**) PEG blocking before (black line) and after (red line) incubation in 100% human serum. (**C**) SWV curves of AuE modified only with the aptamer and challenged it with 10 nM ST spiked in 50% human serum.

One persistent challenge during the implementation of practical applications for newly developed biosensors is severe nonspecific protein adsorption in complex biological matrices. To assess the antifouling ability of the constructed aptasensor, EIS responses of PEG and MCH (common blocking molecules in biosensor fabrication) blocking interfaces were tested before (black lines) and after (red lines) incubation in 100% human serum samples. As shown in Figure 7A, impedance increments of 20% were observed in the Nyquist plot of MCH decorated sensing surfaces, indicating non-negligible protein adsorption on the electrode surface. In contrast, PEG-coated interfaces effectively reduced the unspecific adsorption, and only a 2.7% impedance change was observed after incubation in human serum, Figure 7B. Moreover, SWV responses of aptasensors were also tested after incubation in various diluted human serum samples. It can be seen from Figure S3 that the SWV current response varied with an amplitude of only 6.25% after soaking in 50% diluted serum solution. More importantly, when the PEG mediating aptasensor was incubated into an undiluted human serum sample, the current intensity remained at ~90% of its initial value. The exceptional antifouling property of the PEG monolayer originates from the low gain of adhesion enthalpy and the associated loss of entropy due to steric repulsion during the protein interaction process, which can significantly prevent nonspecific adsorption in complex biological media. The same antifouling performance of PEG has been previously characterized and reported by our group with an aptasensor for malaria detection in blood samples [49]. Moreover, we have also tested the sensor composed of AuE modified only with the S2 aptamer and challenged it with 10 nM ST spiked in human serum. The results obtained for this sensor show only small or inconsistent signals (Figure 7C), in contrast to the data obtained for the sensor with PEG blocking (Table 1). The signal degradation can be ascribed to interfering interactions between aptamer and uncovered electrode areas as well as unspecific adsorption of matrix proteins on the same. Those results further indicated the necessity of the PEG blocking agent for the fabrication of reliable ST sensors that can be operated in complex biological samples.

To further test the potential practical performance of the developed ST aptasensor, recovery experiments were performed in 50% human serum solution by the standard addition method, Table 1. The recovery ranged from 95.4 to 101.4%, with RSD between 3.1 and 4.3%. Besides human serum, also cerebrospinal fluid is a valuable test sample for ST testing. It is found in the CNS and its composition is routinely examined to diagnose various neurological diseases. To verify the versatility of this biosensor, ST detection was conducted in aCSF containing 45 mg dL^−1^ human serum albumin. Our data showed promising conformance of 98.2–103.5% recovery with RSD from 4.8% to 8.7 %, Table 2. From these recovery values, it can be concluded that the biosensor with truncated aptamer demonstrated an assaying performance that complies with the demands of the lab and clinical testing in complex matrices such as human serum and protein-loaded aCSF samples.

## 4. Conclusions

In summary, an electrochemical aptasensor with enhanced antifouling ability was developed for sensitive ST detection by monitoring the SWV current changes. To obtain satisfactory performance, the stem-loop structure of the proposed ST aptamer was truncated and optimized to achieve a proper stem length for enhanced electrochemical signal output. Furthermore, PEG-blocking molecules significantly enhanced the antifouling capabilities of the constructed sensing interface without affecting target-binding performance. Combining the excellent antifouling property of PEG and the high specificity of the aptamer, the developed aptasensor exhibited a wide detection range and a very low LOD for ST. In addition, the proposed aptasensor showed high selectivity and stability. It can also be used for assays targeting ST in complex biological media such as human serum and aCSF. In this work, we essentially optimized all parts of a previously reported aptasensor and distinctly improved the sensing performance and matrix resistance of the electrochemical ST biosensor to pave the way for its direct utilization in in vivo neurotransmitter detection.

## Figures and Tables

**Table 1:** The recovery experiments in 50% human serum (*n* = 3).

Samples Added	Found	Recovery	RSD
0.1 nM	0.101 nM	101.4%	4.3%
10 nM	9.54 nM	95.4%	3.9%
100 nM	100.9 nM	100.9%	3.1%

**Table 2:** The recovery experiments in aCSF (*n* = 3).

Samples Added	Found	Recovery	RSD
1 nM	0.98 nM	98.2%	5.6%
10 nM	10.35 nM	103.5%	4.8%
100 nM	99.20 nM	99.2%	8.7%

## Data Availability

The data presented in this study are available on request from the corresponding author.

## Supplementary Materials

The following supporting information can be downloaded at: https://www.mdpi.com/article/10.3390/bios13090881/s1, Figure S1: Determination of the surface density by chronocoulometric measurements in 10 mM Tris buffer and 50 mM RuHex; Figure S2: Optimization of the target incubation time; Figure S3: SWV responses of AuE with PEG blocking after incubation in different concentrations of human serum samples; Table S1: Performance comparison of the proposed electrochemical aptasensor with other serotonin sensors.
