# Supplementary material for: Truncated Electrochemical Aptasensor with Enhanced Antifouling Capability for Highly Sensitive Serotonin Detection
Source: Biosensors (Basel). 2023 Sep 11;13(9):881. doi: 10.3390/bios13090881 (PMC10527390; doi:10.3390/bios13090881)
Supplement: Supplementary file 1 [file biosensors-13-00881-s001.zip › biosensors-2567928-SI.pdf]

Electronic Supplementary Material (ESI)

Truncated Electrochemical Aptasensor with Enhanced Antifouling Capability for Highly Sensitive Serotonin Detection

Ziheng Hu [1,2], Ruifeng Zhu [1], Gabriela Figueroa-Miranda [1], Lei Zhou [1], Lingyan Feng [3], Andreas Offenhäusser [1] and Dirk Mayer [1,*]

[1] Institute of Biological Information Processing, Bioelectronics (IBI-3), Forschungszentrum Jülich GmbH, 52428 Jülich, Germany; z.hu@fz-juelich.de (Z.H.); r.zhu@fz-juelich.de (R.Z.); g.figueroa.miranda@fz-juelich.de (G.F.-M.); leizhou1@outlook.com (L.Z.); a.offenhaeusser@fz-juelich.de (A.O.)
[2] Faculty I, RWTH Aachen University, 52062 Aachen, Germany
[3] Department of Materials Genome Institute, and Department of Chemistry, College of Science, Shanghai University, Shanghai 200444, China; lingyanfeng@t.shu.edu.cn
* Correspondence: dirk.mayer@fz-juelich.de

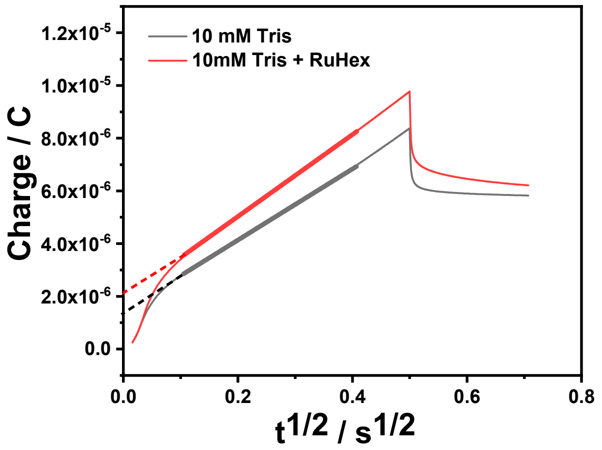

Figure S1. Determination of the surface density by chronocoulometric measurements in 10 mM Tris buffer and 50 mM RuHex.

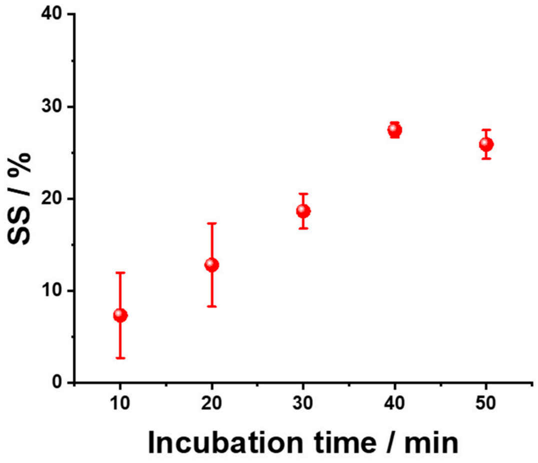

Figure S2. Optimization of the target incubation time.

Table S1. Performance comparison of the proposed electrochemical aptasensor with other serotonin sensors.

Method	Liner range	Detection limit	Ref.
Aptamer/AuNPs	750 nM - 2.5 μM	300 nM	[1]
Aptamer/SWCNT	100 nM - 1 μM	-	[2]
Carbon spheres/GCE	40 μM - 750 μM	700 nM	[3]
AgNP/L-Cys/MXene	500 nM - 150 μM	80 nM	[4]
Aptamer/Gold	1 μM - 100 μM	300 nM	[5]
AMTA/GCE	1 nM - 50 μM	0.013 nM	[6]
Aptamer/PEG	0.1 nM - 1μM	0.14 nM	**This work**

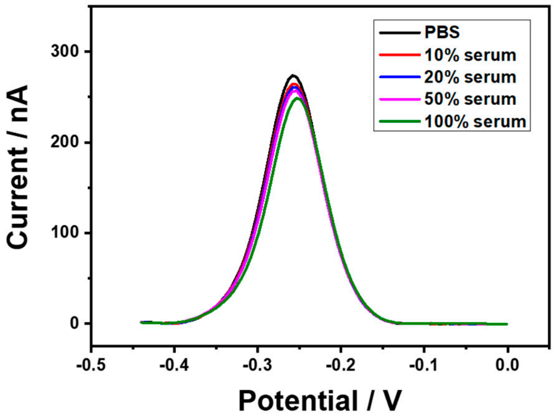

Figure S3. SWV responses of AuE with PEG blocking after incubation in different concentrations of human serum samples.

References

[1] J.L. Chávez, J.A. Hagen, N. Kelley-Loughnane, Fast and Selective Plasmonic Serotonin Detection with Aptamer-Gold Nanoparticle Conjugates, Sensors, 17 (2017), 681.

[2] M. Dinarvand, E. Neubert, D. Meyer, G. Selvaggio, F.A. Mann, L. Erpenbeck, S. Kruss, Near-Infrared Imaging of Serotonin Release from Cells with Fluorescent Nanosensors, Nano Letters, 19 (2019), 6604-6611.

[3] J. Zhou, M. Sheng, X. Jiang, G. Wu, F. Gao, Simultaneous Determination of Dopamine, Serotonin and Ascorbic Acid at a Glassy Carbon Electrode Modified with Carbon-Spheres, Sensors, 13 (2013), 14029-14040.

[4] J. Chen, S. Li, Y. Chen, J. Yang, J. Dong, X. Lu, l-Cysteine-Terminated Triangular Silver Nanoplates/MXene Nanosheets are Used as Electrochemical Biosensors for Efficiently Detecting 5-Hydroxytryptamine, Analytical Chemistry, 93 (2021), 16655-16663.

[5] R. Li, X. Li, L. Su, H. Qi, X. Yue, H. Qi, Label-free Electrochemical Aptasensor for the Determination of Serotonin, Electroanalysis, 34 (2022), 1048-1053.

[6] S.B. Revin, S.A. John, Electrochemical sensor for neurotransmitters at physiological pH using a heterocyclic conducting polymer modified electrode, Analyst, 137 (2012), 209-215.
